# Caste-based differential transcriptional expression of hexamerins in response to a juvenile hormone analog in the red imported fire ant (*Solenopsis invicta*)

**DOI:** 10.1371/journal.pone.0216800

**Published:** 2019-05-20

**Authors:** Chloe Hawkings, Travis L. Calkins, Patricia V. Pietrantonio, Cecilia Tamborindeguy

**Affiliations:** Department of Entomology, Texas A&M University, College Station, Texas, United States of America; Durham University, UNITED KINGDOM

## Abstract

The reproductive ground plan hypothesis proposes that gene networks regulating foraging behavior and reproductive female physiology in social insects emerged from ancestral gene and endocrine factor networks. Expression of storage proteins such as vitellogenins and hexamerins is an example of this co-option. Hexamerins, through their role modulating juvenile hormone availability, are involved in caste determination in termites. The genome of the fire ant (*Solenopsis invicta*) encodes four hexamerin genes, *hexamerin-like* (LOC105192919, hereafter called *hexamerin 1)*, *hexamerin* (LOC105204474, hereafter called *hexamerin 2)*, *arylphorin subunit alpha-like*, and *arylphorin subunit beta*. In this study, a phylogenetic analysis of the *S*. *invicta* hexamerins determined that each predicted protein clustered with one of the orthologous *Apis mellifera* hexamerins. Gene expression analyses by RT-qPCR revealed differential expression of the hexamerins between queens and workers, and between specific task-allocated workers (nurses and foragers). Queens and nurses had significantly higher expression of all genes when compared to foragers. *Hexamerin 1* was expressed at higher levels in queens, while *hexamerin 2* and *arylphorin subunit beta* were expressed at significantly higher levels in nurses. *Arylphorin subunit alpha-like* showed no significant difference in expression between virgin queens and nurses. Additionally, we analyzed the relationship between the expression of hexamerin genes and S-hydroprene, a juvenile hormone analog. Significant changes in hexamerin expression were recorded in nurses, virgin queens, and foragers 12 h after application of the analog. *Hexamerin 1* and *arylphorin subunit alpha-like* expression were significantly lower after analog application in virgin queens. In foragers, *hexamerin 2* and *arylphorin subunit beta* were significantly lower after analog application, while in nurses expression of all genes were significantly lower after analog application. Our results suggest that in *S*. *invicta* hexamerin genes could be associated with reproductive division of labor and task-allocation of workers.

## Introduction

The reproductive ground plan hypothesis proposes that the gene networks that regulate foraging behavior and reproductive female physiology in social insects emerged from ancestral networks of genes and endocrine factors [1,2,3]. Examples of this co-option of gene networks to regulate division of labor have been shown in several social hymenopteran species [4,5,6,7]. One of the genes recognized for its co-optive role is vitellogenin (Vg), which encodes a soluble yolk protein precursor involved in the production of eggs in oviparous species. In social insects, Vg expression in the queen is linked to its conventional role in reproduction, while in the workers it is linked to social organization, task allocation and age polyethism [8,9,10,11,12]. In *Apis mellifera*, Vg levels decrease as the workers transition from nursing to foraging tasks [8], and silencing of the Vg gene in nurses resulted in precocious foraging [13]. Similar studies conducted in several ant species have also identified a potential role of Vg in task allocation and colony organization based on the patterns of expression in task allocated insects or in response to changes in social context. For instance, *Pogonomyrmex barbatus* nurses express high levels of Vg transcript which decrease as the individual transitions into foraging tasks [14]. Similarly, in *Ectatomma tuberculatum*, production of Vg is related to the worker age: workers produce high levels of Vg at the beginning of their adult life when they conduct tasks associated with the brood inside the nest, while older workers which perform tasks outside the nest do not produce Vg [15].

In insects, juvenile hormone (JH) is a developmental hormone during the immature stages, and it can play a gonadotropic role promoting reproduction in adults [16]. The behavioral transition of workers, and the dominance position and reproductive status of hymenopteran queens have also been linked to JH titers [17,18,19,20,21,22], suggesting that JH may play a role in the expression of proteins that will change the behavior or task of an individual [22]. The role of JH in social insect age polyethism has been extensively studied in *A*. *mellifera* [18,23,24]. In this species, JH level is typically low in nurses, and increases as the workers transition into performing foraging tasks. JH is also involved in age polyethism in ants. For example, *Pogonomyrmex californicus*, *Myrmicaria eumenoides* and *Harpegnathos saltator* foragers have higher JH titers than the workers performing in-nest tasks [25,26,27]. Furthermore, in some ant species, a correlation between JH titer and Vg was identified. For example, Vg titer was down-regulated in *E*. *tuberculatum* sterile workers following topical application of JH [21].

In addition to Vg, hexamerins, a second family of storage proteins, have been also associated with social organization. Hexamerins, also known as larval storage proteins, are synthesized in the fat body, and are secreted and accumulate in the hemolymph of larvae. Later in development, they are reabsorbed into the fat body, broken down, and incorporated into adult tissues during the pupal molt [28]. In termites, hexamerins are involved in the regulation of caste determination because silencing of hexamerins results in a higher proportion of soldiers [29,30]. This latter function may be through an interaction of hexamerins with JH signaling [31]. In *Polistes metricus*, a social wasp, insects that will emerge to become gynes have higher Hexamerin 1 protein levels compared to those that will become workers [32]. Furthermore, several studies reported differences in hexamerin expression between castes of social Hymenoptera [33,34,35,36]. Specifically, in *A*. *mellifera*, two hexamerin genes are expressed in the adult fat body in a caste- and sex-specific manner, with higher expression in workers than in queens [37]. In ants, these proteins accumulate during the alate virgin period of the adult queen life stage and may serve as amino acid storage [38]. In some species, this storage is critical to the claustral period of colony formation; hexamerins allow queens to produce the first generation of workers without having to leave the nest to forage [38,39].

Investigating the expression and function of storage proteins in *Solenopsis invicta* is of particular interest because this species displays extreme reproductive division of labor between two basic caste forms. The worker caste is composed of polymorphic sterile females responsible for the tasks that support the growth and maintenance of the colony [40], while the reproductive adults are queen(s) and drones, which are responsible for producing the offspring. In a previous study, we investigated the expression of Vgs in the worker caste of *S*. *invicta* and we identified differences in the expression of Vg1 among workers performing different tasks [10]. Further, in contrast to worker bees, none of the four Vg genes were regulated in workers by topical application of the JH-analog S-hydroprene [10]. In a different study, we have shown that when comparing brain transcriptomes of alate virgin and dealate mated queens, the expression of two hexamerins, *hexamerin-like* (LOC105192919, hereafter called *hexamerin 1*) and *arylphorin subunit alpha-like* (LOC105192898), is significantly reduced in brains of mated queens [41]. In the same transcriptome analysis, no differences in expression were observed for the other two hexamerin genes, *hexamerin* (LOC105204474, hereafter called *hexamerin 2*) and *arylphorin subunit beta* (LOC105192897). Moreover, the topical application of the JH analog S-hydroprene resulted in decreased expression of *hexamerin 1* in whole bodies of virgin queens [41]. However, the effect of S-hydroprene on the expression of the other hexamerin genes in queens and on the expression of all hexamerins in adult workers was not determined.

Despite the knowledge about the involvement of storage proteins in caste determination in social insects, the expression and roles of hexamerin genes in *S*. *invicta* has not yet been investigated. In particular, knowledge gaps exist regarding the relationship between hexamerin expression and JH, a key regulator of social organization. In this species, while JH has retained its gonadotropic role in the queen [42], it is still unknown if JH is involved in worker age polyethism. Similarly, whether hexamerin expression is regulated by JH in workers or the interplay of JH and hexamerins in the regulation of worker tasks in *S*. *invicta* has not been investigated. Thus, in this study, we evaluated the expression of the four hexamerin genes in *S*. *invicta* workers and queens, and investigated whether those genes were regulated by a JH analog in a caste- or task-specific manner.

## Materials and methods

### Insect colonies

Polygyne colonies of *S*. *invicta* were collected in College Station, Brazos County, Texas, from July to September of 2017 and maintained in the Department of Entomology at Texas A&M University, College Station, Texas, as laboratory colonies in plastic containers (27 x 40 x 9 cm). The inside of these containers was coated with Fluon to prevent ant escape (Insect-a-slip, Bioquip products, CA). The colonies were kept at 27± 2°C in a 12:12 hour dark-light photoperiod. Colonies were provided with half-filled water tubes, capped with cotton as a damp nesting area, and fed daily with both 20% honey solution and crickets (*Acheta domestica*) as a protein source. Water was provided *ad libitum*. Colonies contained mated queens (dealate), virgin queens (alate), drones, polymorphic workers and brood (eggs, larvae and pupae). Mated queens for gene expression analyses were collected immediately after a mating flight in College Station, Texas in May 2018. These mated queens were kept as previously described in a container with half-filled water tubes, capped with cotton as a damp nesting area for approximately one week after the first batch of eggs were laid.

### Phylogenetic analysis

*Apis mellifera*, *Nasonia vitripennis*, *Camponotus floridanus*, *Harpegnathos saltator*, *Acromyrmex echinatior*, *Atta colombica*, *and Lasius niger* hexamerin proteins were identified using Blastp searches or mining the respective genomes. The protein sequences were downloaded in fasta format and aligned using MAFFT v.7 [43] using the amino acid alignment default parameters. The alignments were visually assessed using Mesquite v. 3.03 [44] and the sequences were manually trimmed. The phylogenetic tree was reconstructed by Bayesian inference (Mr. Bayes v 3.2.3) [45] on the CIPRES supercomputer [46] with four runs of a mixed amino acid model for 1,000,000 generations, and a 10% burnin. Convergence of the runs were assessed in Tracer 1.6. The consensus tree was visualized in FigTree version 1.4.2 (http://tree.bio.ed.ac.uk/software/figtree/) and rooted using the termite *Reticulitermes flavipes* hexamerin proteins as the outgroup.

### Classification and selection of ants

Virgin queens (alate) were selected based on the presence of wings. Mated queens (dealate) were selected if they had laid eggs. The workers were classified based upon their head width [47], and for this study only medium-size workers were used (head width between 0.73 and 0.92 mm), which are the intermediate size workers within the colony. Foraging workers were individuals in the foraging arena who were actively interacting with food resources outside of the nest. Nurses were individuals inside the nest that were actively interacting with the brood (any immature developmental stage). All individuals were of unknown age.

### Expression analysis of hexamerins in workers and queens

Gene expression analyses were conducted using pools of ten individual workers or of five individual queens to obtain sufficient RNA. Individuals in each pool were from the same colony; each pool was obtained from a different colony. Workers were pooled according to the task performed (nursing or foraging). Pooled alate virgin and mated queens were of unknown age. Pools were flash-frozen in liquid nitrogen upon collection and stored at -80°C until gene expression analysis.

Insects were ground in liquid nitrogen to a fine powder using a mortar and pestle. Total RNA extractions were conducted using Trizol reagent (Invitrogen, Carlsbad, CA) following the manufacturer’s protocol. Purification of RNA was performed using the Direct-zol microprep kit (Zymo Research, Irvine, CA) for clean-up of the samples. RNA was resuspended in 20 μl of nuclease-free water. Genomic DNA was eliminated using the Turbo DNAse kit (Ambion, Waltham, MA) following the manufacturer’s protocol. Total RNA was quantified using the Infinite 200 PRO NanoQuant (Tecan, Männedorf, Switzerland), and its integrity was assessed using gel electrophoresis, visualized on a 2% agarose gel stained with ethidium bromide.

For gene expression analyses, RT-qPCR reactions were performed using the SensiFAST SYBR Hi-ROX One Step kit (Bioline, Taunton, MA) following the manufacturer’s protocol. All reactions contained 50 ng of total RNA, 250 nM of forward and 250 nM of reverse primers [41], 2X SensiFAST SYBR Hi-ROX One-Step Mix, reverse transcriptase, and RiboSafe RNAse inhibitor; the volume was adjusted with nuclease-free water to 10 μl. Primers are listed in S1 Table. The thermocycler parameters were set to 45°C for 10 min, then 95°C for 2 min and 40 cycles of 95°C for 5 sec and 60°C for 30 sec. RT-qPCR was performed using the Applied Biosystems QuantStudio 6 Flex Real-Time PCR System (Thermo Fisher Scientific, Waltham, MA) according to manufacturer’s instructions. Each reaction was performed in duplicate, and negative controls for each reaction were included. Primer specificity was monitored with a melting curve analysis using the QuantStudio software V1.3 (Thermo Fisher Scientific). Relative expression of each hexamerin gene was determined using the ΔΔCt method [48] by normalizing the level of each hexamerin transcript to the internal control gene, *rp18* which is stable among different *S*. *invicta* castes [49]. For each gene expression assay, six independent biological replicates were analyzed; one replicate consisted of one pool of ants.

### Juvenile hormone analog study

Alate virgin queens and medium-size workers (nurses and foragers) were collected and treated topically with 1 μl of the JH analog, S-hydroprene (analytical standard catalogue number 46426, Sigma-Aldrich, St. Louis, MO) (25 ng/μl dissolved in acetone) with a pipette on the dorsal surface of the abdomen, or with 1 μl of acetone on the abdomen once during the assay. The chosen S-hydroprene dose was used in previous experiments on ants [10,41]. Fresh solutions of S-hydroprene were prepared for each biological replicate. Also included for each biological replicate was a non-treatment control group, which consisted of ants that were handled similarly to the treatment group but to which no solution was topically applied. Following treatment, ants from each treatment group were caged together in separate containers and placed within their original colony (S1 Fig). Cages (15 ml tube) were enclosed with very fine nylon mesh, which separated each group from the rest of the colony. Six replicates were conducted using six independent colonies. As expected, only the S-hydroprene treatment induced dealation of the alate virgin queens: 100% of the treated queens dealated while none of the non-treatment or acetone-treated virgin queens dealated. Therefore while the fine mesh separated individuals from the rest of the colony, it did not isolate them nor prevented contact with the other insects in the colony.

Ten medium-size foragers, medium-size nurses, or alate virgin queens from each treatment group were collected twelve hours after treatment, pooled by treatment, flash-frozen, and stored at -80°C until gene expression analyses. Total RNA extractions from each pool and RT-qPCR analyses were conducted as described before.

### Statistical analysis

Data are reported as the mean ± the standard error of the mean (SEM). Data were log transformed and the normality of the log CT was verified after transformation. Statistical analyses were conducted using the one-way ANOVA test and Tukey-Kramer Post Hoc with JMP Version 13 (SAS Institute Inc., Cary, NC, 1989–2017) and the estimated *p*-values were considered significant when below the 0.05 threshold.

## Results

### Phylogenetic analysis

**Fig 1:**
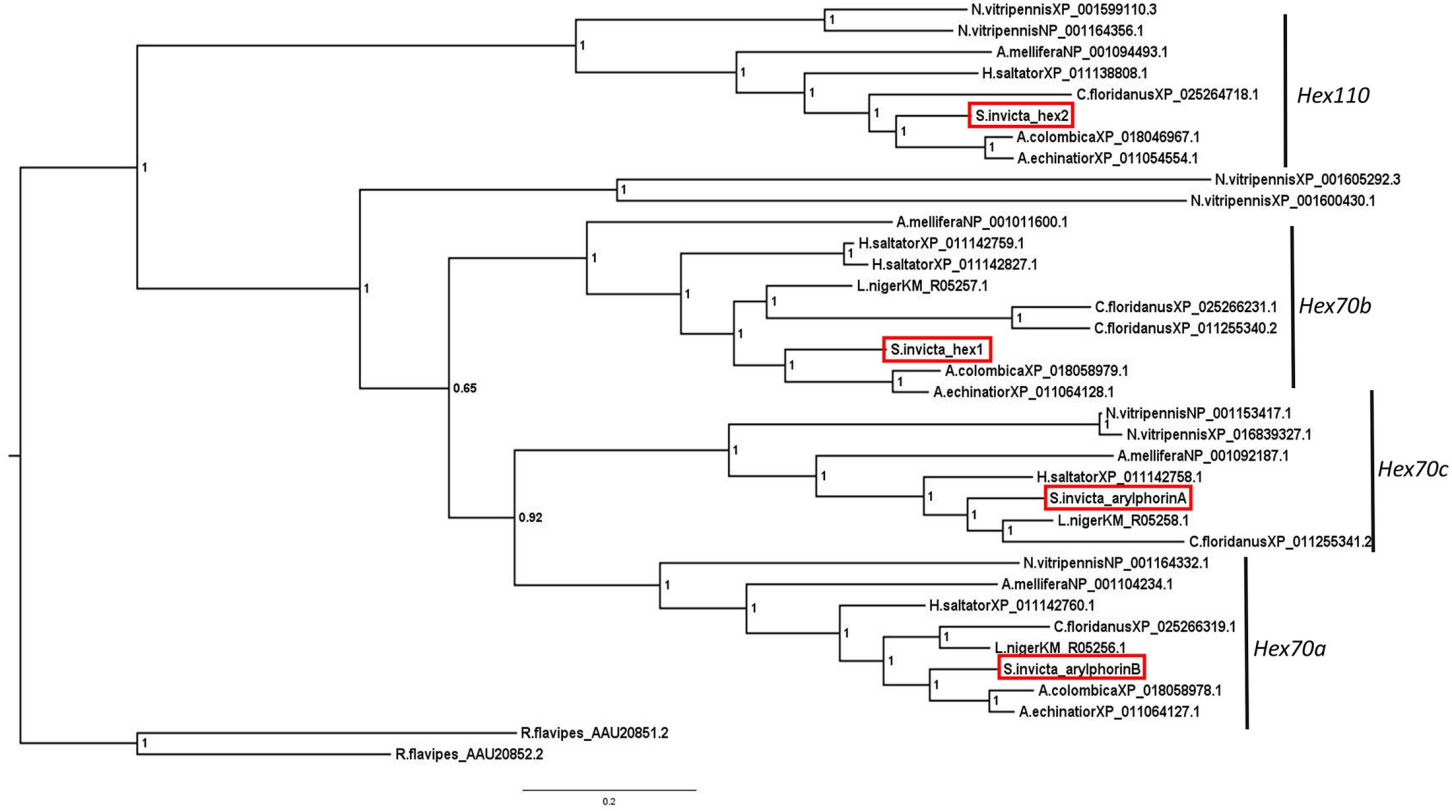
Phylogenetic analysis of *S*. *invicta* and other hymenopteran hexamerin candidate proteins under Bayesian inference. Node value posterior probability was rounded to two significant figures. Scale bar indicates branch length. The two hexamerins of the termite *R*. *flavipes* were used as the outgroup. The four clade names represent the four *A*. *mellifera* hexamerin proteins. The hexamerins of *S*. *invicta* (boxed in red) clustered as follows: Hexamerin 1 (XP_025995150.1) with Hex 70b, Hexamerin 2 (XP_011171862.1) with Hex 110, Arylphorin subunit alpha-like (XP_011155485.1) with Hex 70c, and Arylphorin subunit beta (XP_011155484.1) with Hex 70a. Alignment of the *S*. *invicta* hexamerin proteins can be found in supplementary information (S2 Fig).

A Bayesian analysis was conducted to evaluate the phylogenetic association of *S*. *invicta* hexamerins with other hymenopteran hexamerins (Fig 1). Strong support for nodes was found on the phylogeny. Most nodes had 100% posterior probability value; two nodes did not but achieved 92% and 65% support. The obtained phylogenetic tree has two major hexamerin clades as in [50] which analyzed *N*. *vitripennis* hexamerins.

The smaller hexamerin clade included the largest *S*. *invicta* hexamerin, Hexamerin 2, (S. invicta_hex2 in Fig 1) which clustered more closely with hexamerins from four ant species, *H*. *saltator* XP_011138808.1, *A*. *colombica* XP_018046967.1, *A*. *echinatior* XP_011054554.1, and *C*. *floridanus* XP_025264718.1 as well as with *A*. *mellifera* NP_001094493.1 (Hex 110), *N*. *vitripennis* NP_001164356.1 and *N*. *vitripennis* XP_001599110.3. Similar to the proteins from these other species, *S*. *invicta* Hexamerin 2 is characterized by a high glutamic acid and glutamine (Glx) content (25.4%) (Table 1). *S*. *invicta* Hexamerin 2 is located in the contig NW_020522001.1 in the *S*. *invicta* genome.

**Table 1:** Composition of the predicted *S*. *invicta* hexamerin proteins: number of residues typically enriched in insect hexamerins [38] and their corresponding percentage (in parenthesis).

Hexamerin ID	N° of aromatic amino acids (%)	Tyrosine (%)	Glx^a^ (%)	Methionine (%)	Leucine (%)
Hexamerin 1 XP_025995150.1 (686 amino acids)	98 (14.29%)	53 (7.73%)	60 (8.75%)	21 (3.06%)	48 (7.00%)
Hexamerin 2 XP_011171862.1 (1027 amino acids)	102 (9.93%)	79 (7.69%)	261 (25.41%)	2 (0.19%)	79 (7.69%)
Arylphorin subunit alpha-like XP_011155485.1 (696 amino acids)	145 (20.83%)	79 (11.3 5%)	55 (7.90%)	27 (3.88%)	58 (8.33%)
Arylphorin subunit beta XP_011155484.1 (696 amino acids)	129 (18.53%)	72 (10.34%)	58 (8.33%)	19 (2.73%)	56 (8.05%)

^a^Glx are glutamic acid and glutamine.

The other three *S*. *invicta* hexamerins, which are adjacent in the *S*. *invicta* genome on the same contig (NW_020521759.1), show a higher degree of similarity among themselves (Fig 1 and Table 2). *S*. *invicta* Hexamerin 1 clustered with *A*. *mellifera* NP_001011600.1 (Hex 70b). Interestingly, *N*. *vitripennis* XP_001605292.3 and XP_001600430.1, which had previously clustered with *A*. *mellifera* Hex 70b [50] were not in this cluster. *S*. *invicta* Arylphorin subunit alpha-like clustered with *A*. *mellifera* NP_001092187.1 (Hex 70c), and two *N*. *vitripennis* proteins, NP_001153417.1 and XP_016839327.1. Finally, *S*. *invicta* Arylphorin subunit beta clustered with *A*. *mellifera* NP_001104234.1 (Hex 70a) and *N*. *vitripennis* NP_001164332.1. Each of these *S*. *invicta* proteins were more similar to the ant hexamerins than to the *A*. *mellifera* or *N*. *vitripennis* hexamerins. Both *S*. *invicta* arylphorin proteins have high aromatic amino acid content (Table 1). Hexamerin 1 failed to meet the 15% aromatic amino acid criterion to be considered an arylphorin (Table 1). None of the *S*. *invicta* hexamerins are leucine-rich (>10% leucine) or methionine-rich (> 4% methionine) proteins [51].

**Table 2:** Percentage sequence identities and similarities among the *S*. *invicta* hexamerins determined by BlastP analyses. Identities are shown above, similarities below the diagonal line represented by shaded cells.

	Arylphorin subunit alpha-like	Hexamerin 1	Arylphorin subunit beta	Hexamerin 2
Arylphorin subunit alpha-like	-	44	50	31
Hexamerin 1	62	-	46	33
Arylphorin subunit beta	67	66	-	31
Hexamerin 2	53	54	51	-

### Expression of hexamerins in workers and queens

**Fig 2:**
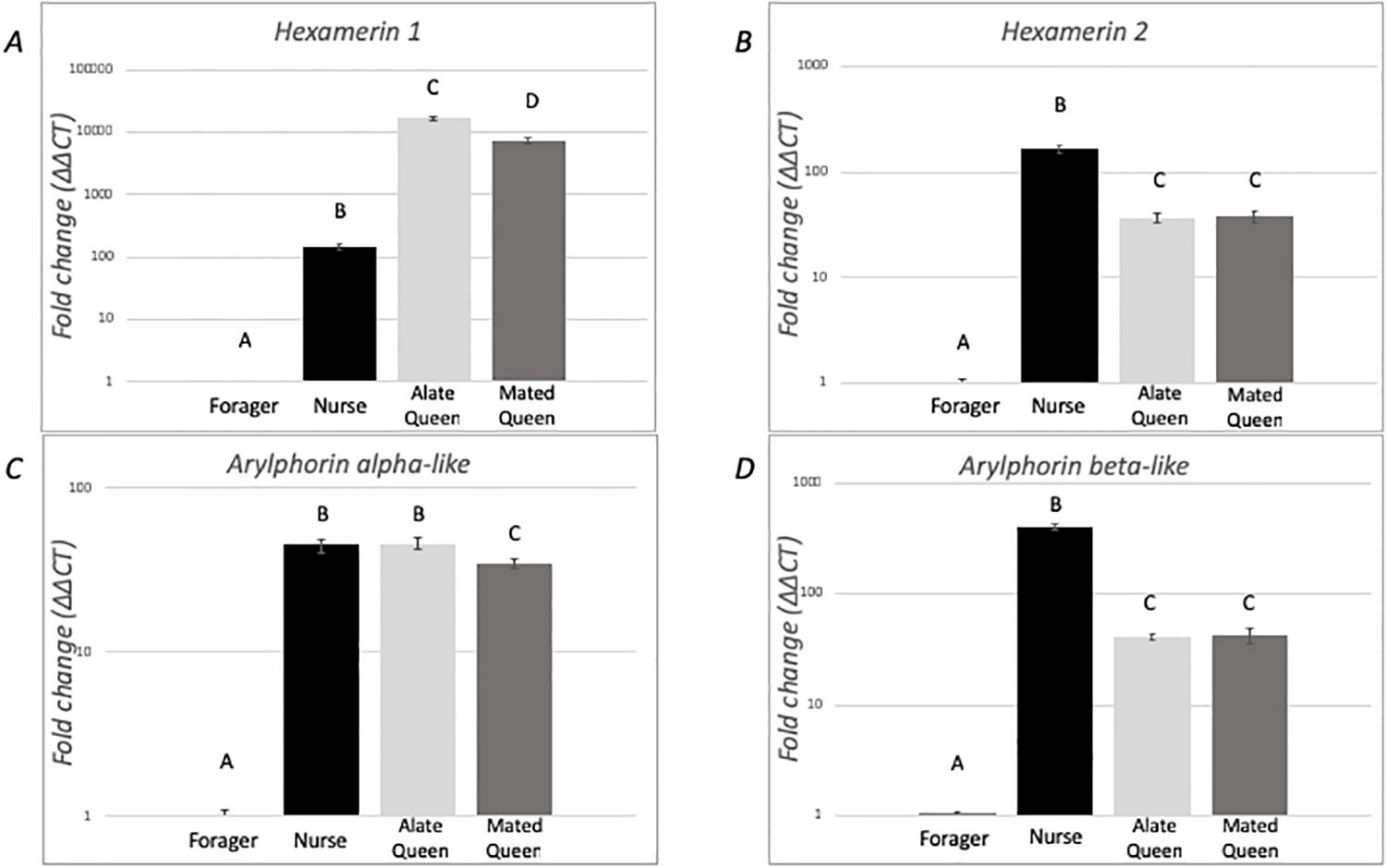
Expression analysis of hexamerin transcripts among medium-sized foragers, medium-sized nurses, alate virgin queens, and dealate mated queens. Relative expression levels of (A) *hexamerin 1*, (B) *hexamerin 2*, (C) *arylphorin subunit alpha-like*, and (D) *arylphorin subunit beta* transcripts. Gene expression was quantified using RT-qPCR and analyzed using the ΔΔCt method. For each gene, mRNA expression level was normalized relative to *rp18*. Bars represents mean ± SEM fold change relative to foragers (*n* = 6). Different letters indicate significant differences in gene expression as determined by one-way ANOVA with Tukey’s post hoc analyses (*p* < 0.05). The fold change in the Y-axis is in log-scale.

The patterns of expression of *hexamerin 1*, *hexamerin 2*, *arylphorin subunit alpha-like* and *arylphorin subunit beta* transcripts were obtained for alate virgin queens, dealate mated queens, medium-size foragers, and medium-size nurses. Significant differences were found in the expression of all genes. Foragers had the lowest expression of all tested genes (Fig 2). For *hexamerin 1* (Fig 2A), significant differences were found among the four groups (F = 2270.19, df = 3, *p* < 0.0001). Alate virgin queens had the highest level of expression, significantly higher than the other three groups, while dealate mated queens had significantly higher levels of expression compared to nurses and foragers, and nurses had significantly higher levels than foragers. Similar expression profiles were obtained for *hexamerin 2* (Fig 2B) (F = 424.06, df = 3, *p* < 0.0001) and *arylphorin subunit beta* (Fig 2D) (F = 713.60, df = 3, *p* < 0.0001). Nurses had the highest levels of expression. Alate virgin and dealate mated queens had similar levels of expression, which was significantly higher than foragers. For *arylphorin subunit alpha-like* (Fig 2C), significant differences were found among groups (F = 671.87, df = 3, *p* < 0.0001). No significant difference in expression was found between nurses and alate virgin queens. Dealate mated queens had significantly lower expression than the nurses and alate virgin queens, but significantly higher than foragers.

### Expression of hexamerins in medium-size foragers following topical application of S-hydroprene

**Fig 3:**
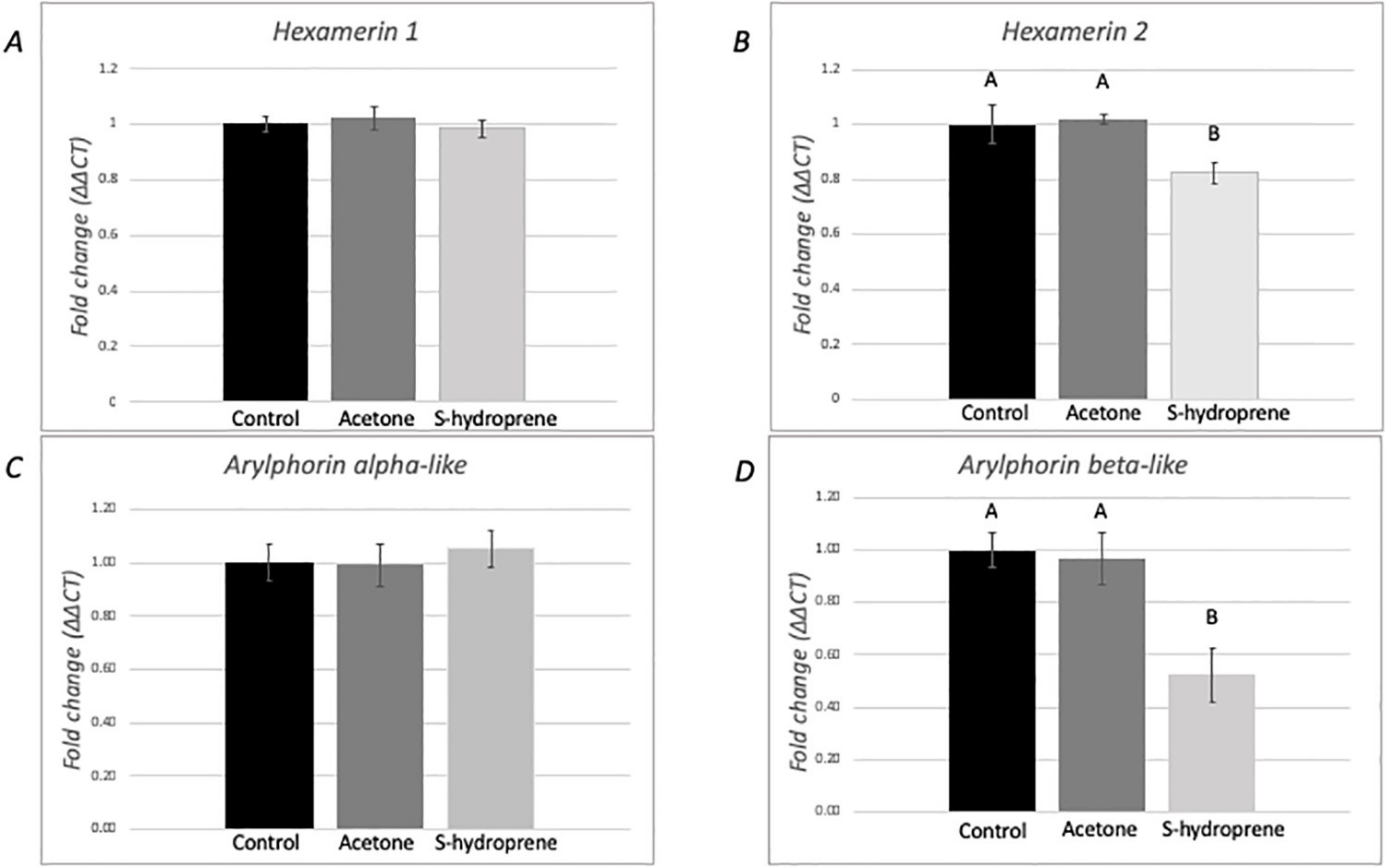
Expression analysis of hexamerin transcripts in medium-sized foragers 12 h following topical application of S-hydroprene. Relative expression of (A) *hexamerin 1*, (B) *hexamerin 2*, (C) *arylphorin subunit alpha-like*, and (D) *arylphorin subunit beta* transcripts. Gene expression was quantified using RT-qPCR and analyzed using the ΔΔCt method. For each gene, mRNA expression level was normalized relative to *rp18*. Bars represents mean ± SEM fold change relative to the untreated control (*n* = 6). Statistical relationships between groups were assessed using one-way ANOVA. Significant differences in gene expression (*p* < 0.05) were determined for *hexamerin 2* and *arylphorin subunit beta*. For those genes, different letters indicate significant differences in gene expression as determined with Tukey post hoc analyses.

The expression of the four hexamerin genes was evaluated from pools of ten individual medium-size foragers 12 hours after S-hydroprene application (Fig 3). No significant differences in expression were found for *hexamerin 1* (Fig 3A) and *arylphorin subunit alpha-like* (Fig 3C) among the treatment groups (*p* > 0.05). However, significant differences in expression were identified for *hexamerin 2* (F = 17.48, df = 2, *p* = 0.003, Fig 3B) and *arylphorin subunit beta* (F = 77.96, df = 2, *p* < 0.0001, Fig 3D). In both cases, expression was significantly lower in the S-hydroprene treatment when compared to both control treatments (non-treatment and acetone).

### Expression of hexamerins in medium-sized nurses following topical application of S-hydroprene

**Fig 4:**
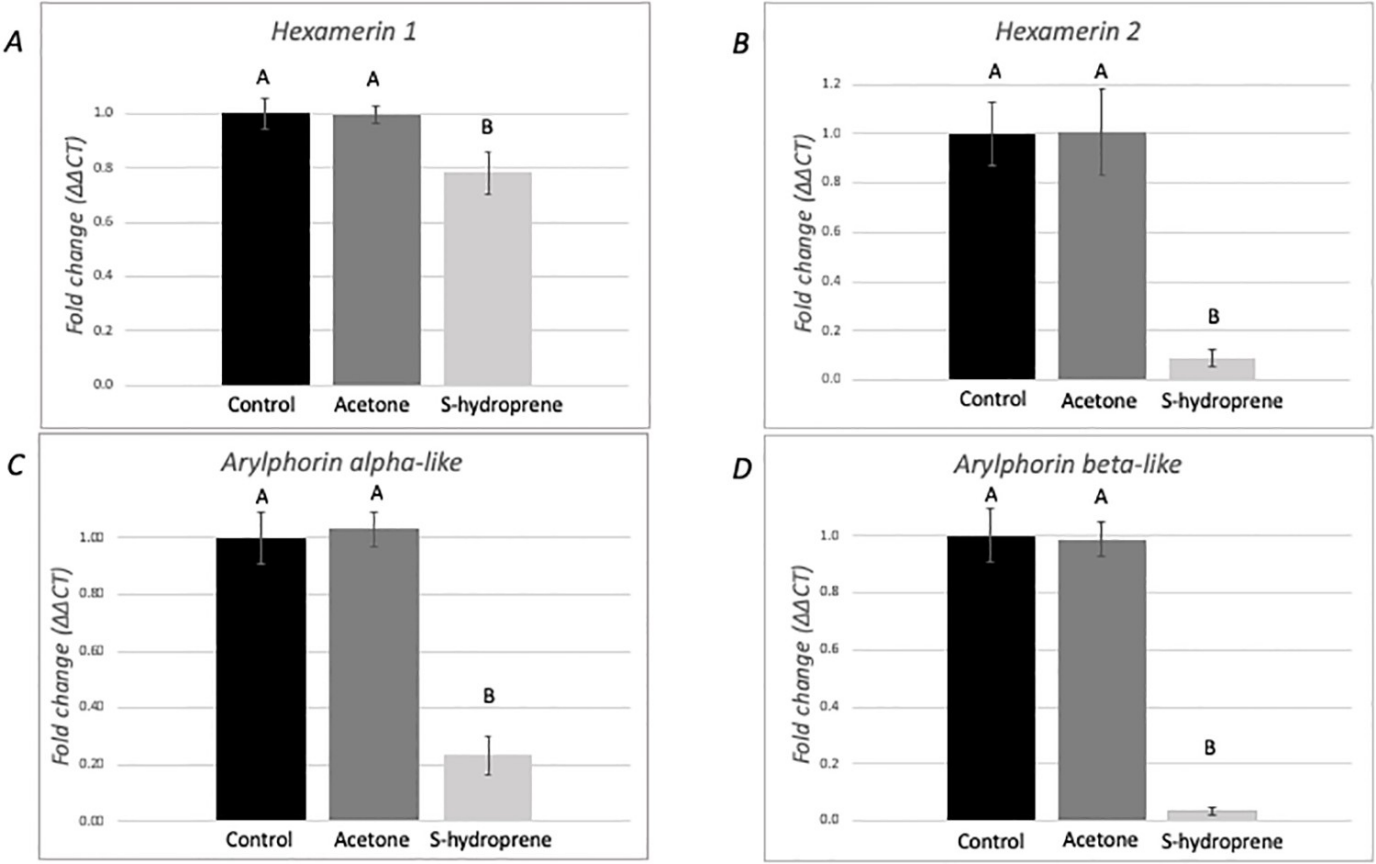
Expression analysis of hexamerin transcripts in medium-sized nurses 12 h after topical application of S-hydroprene. Relative expression of (A) *hexamerin 1*, (B) *hexamerin 2*, (C) *arylphorin subunit alpha-like*, and (D) *arylphorin subunit beta* transcripts. Gene expression was quantified using RT-qPCR and analyzed using the ΔΔCt method. For each gene, mRNA expression level was normalized relative to *rp18*. Bars represents mean ± SEM fold change relative to untreated control (*n* = 6). Different letters indicate significant differences in gene expression as determined by one-way ANOVA with Tukey’s post hoc analyses (*p* < 0.05).

The pattern of expression of the four hexamerin genes was evaluated from pools of ten individual medium-size nurses 12 hours after S-hydroprene application (Fig 4). In this case, the expression of all genes was significantly reduced in the S-hydroprene treatment group compared to the control treatments: *hexamerin 1* (F = 4.79, df = 2, *p* = 0.025, Fig 4A); *hexamerin 2* (F = 17.48, df = 2, *p* = 0.0001, Fig 4B); *arylphorin subunit alpha-like* (F = 36.88, df = 2, *p* < 0.0001, Fig 4C), and *arylphorin subunit* (F = 77.96, df = 2, *p* < 0.0001, Fig 4D).

### Expression of hexamerins in alate virgin queens following topical application of S-hydroprene

**Fig 5:**
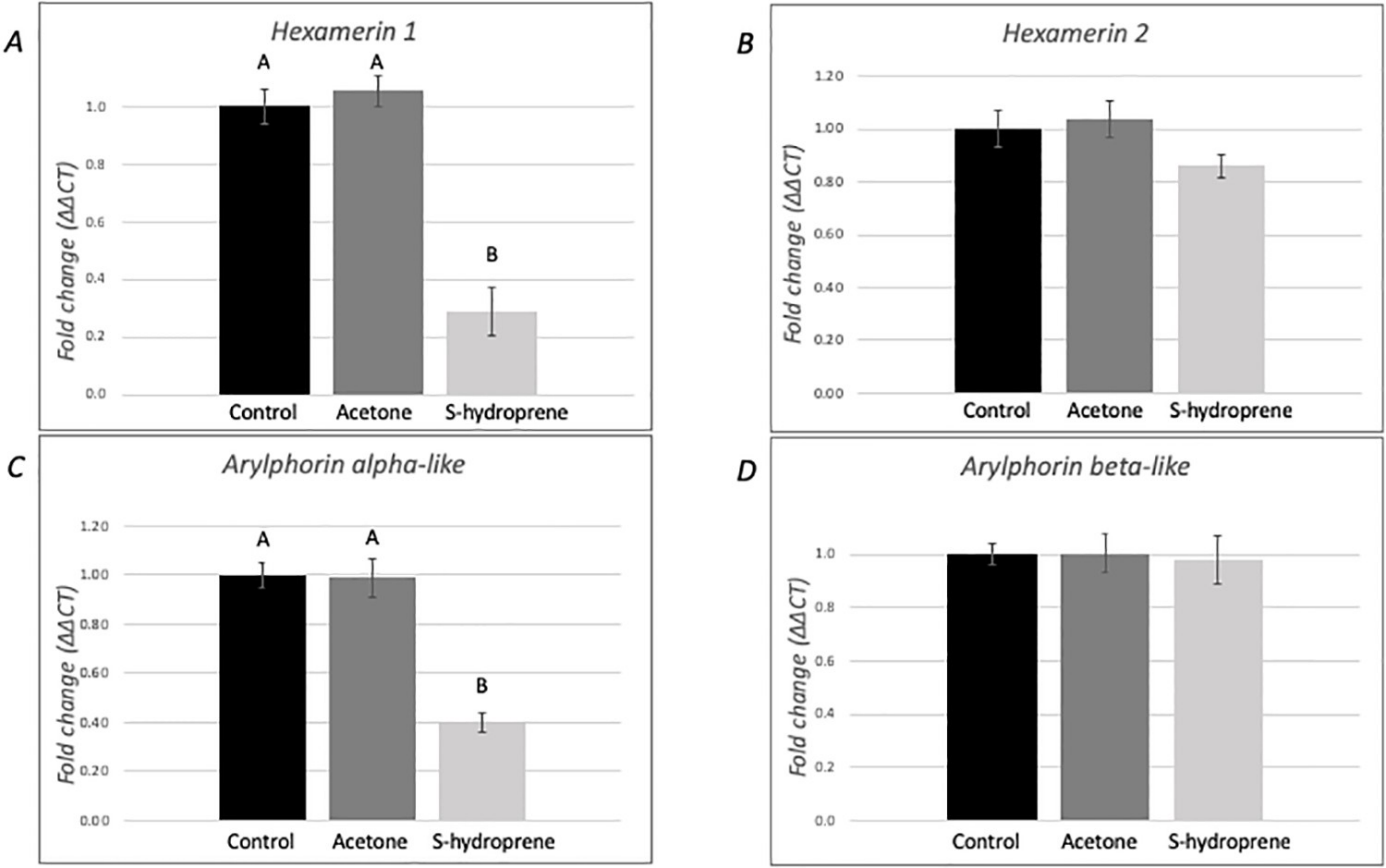
Expression analysis of hexamerin transcripts in alate virgin queens 12 h following topical application of S-hydroprene. Relative expression of (A) *hexamerin 1*, (B) *hexamerin 2*, (C) *arylphorin subunit alpha-like*, and (D) *arylphorin subunit beta* transcripts. Gene expression was quantified using RT-qPCR and analyzed using the ΔΔCt method. For each gene, mRNA expression level was normalized relative to *rp18*. Bars represents mean ± SEM fold change relative to untreated control (*n* = 6). Significant differences in gene expression (*p* < 0.05) were determined for *hexamerin 1* and *arylphorin subunit alpha-like*. For those genes, different letters indicate significant differences in gene expression as determined with Tukey post hoc analyses.

The pattern of expression of the four hexamerin genes was evaluated from pools of ten virgin queens 12 hours after S-hydroprene application. No differences in the expression of *hexamerin 2* (Fig 5B) or *arylphorin subunit beta* (Fig 5D) were found among the treatments (*p* > 0.05), while differences were found for *hexamerin 1* (F = 40.94, df = 2, *p* = < 0.0001, Fig 5A), and for *arylphorin subunit alpha-like* (F = 33.27, df = 2, *p* = <0.0001, Fig 5C). In both cases, the expression of was significantly lower in the S- hydroprene treatment group than in both control groups.

## Discussion

In holometabolous insects, hexamerin proteins accumulate during larval development [51] and serve as an amino acid source during the pupal and pharate adult stages. Insects encode several hexamerins, and the copy number of hexamerin genes varies within social insects.. Four hexamerin genes are predicted in the *S*. *invicta* genome (LOC105192919, LOC105192898, LOC105204474, and LOC105192897). Based on the phylogenetic analysis, they correspond to each of the four hexamerin types found in *A*. *mellifera* (*hex 70a*, *hex 70b*, *hex 70c*, *hex 110*). This is consistent with previous analyses of hymenopteran genomes which typically harbor at least one copy of each of the four hexamerins commonly identified in *A*. *mellifera* [52]. Interestingly, our search of hexamerins among the chosen ant species failed to identify the homolog of *hex 70c* in the leaf-cutting ants *A*. *colombica* and in *A*. *echinatior* which could be linked with the mutualism established between these species and fungi [52]. In addition to *hex 70c* in the aforementioned ants, a homolog of *hex 110* was not found in the current annotation of *L*. *niger* genome.

In *S*. *invicta* the four hexamerin genes were expressed in queens and in workers. The gene expression of all hexamerins was lower in foragers than in nurses or in queens. *Hexamerin 2* and *arylphorin subunit beta* were expressed at higher levels in nurses than in queens, while *arylphorin subunit alpha-like* was expressed at higher levels in nurses than in mated queens, but not virgin queens. In ants, expression of hexamerin genes in adults, and differences of expression between the worker and queen castes were recorded in *Formica exsecta* [53]. In this species, all hexamerin transcripts are expressed at higher level in emerging queens than in emerging workers, while the opposite pattern is found in older insects because old workers have higher hexamerin transcript expression than old queens (Supporting information in [53]). Moreover, the accumulation of hexamerin 1 and 2 in virgin queens of *Camponotus festinatus* has been associated with claustral colony founding [39]. This is in contrast to *A*. *mellifera* in which the expression of *hex 70a* and *hex 110* genes in queens is relatively low and occurs in the ovaries of egg-laying queens [37,54,55]. Further, these hexamerin genes are the only hexamerins with detectable expression in the *A*. *mellifera* adult worker fat body [37].

It is noteworthy that expression of all four *S*. *invicta* hexamerins was down-regulated in foragers when compared to nurses. This finding is in accordance with transcriptomic results comparing gene expression between *S*. *invicta* nurses and foragers (Supplementary data in [56]). Similarly, in *A*. *mellifera*, the expression of *hex 70a* and of *hex 110* decreased as workers aged, and this decrease corresponded with their transition to foraging [37]. This change in gene expression coincides with the increase in the JH titer as the *A*. *mellifera* workers age. Therefore, the expression of hexamerins in social insects could be associated with the specific task being performed by the worker, with the age of the worker, or result from the interaction of both task and age and other factors. Comparison of the *hex 110* and *hex 70a* expression between queenless *A*. *mellifera* workers showed activation of the expression of both genes in workers with active ovaries compared to those carrying inactive ovaries [57,58]. *S*. *invicta* nurses do not have the potential for reproduction; therefore, the expression of hexamerins in workers of this ant species is likely linked to other functions. As such, nurses could be using hexamerins as reserves to nourish brood and/or in nutritional signaling [29,41]. For instance, *Camponotus festinatus* workers accumulate Hexamerin 1 and Hexamerin 2 proteins when reared in the absence of brood, but not in the presence of larvae [39].

Previously, we had shown that *hexamerin 1* expression in *S*. *invicta* virgin queens was down-regulated following topical application of a JH analog [41]. Here, we evaluated the expression of the four *S*. *invicta* hexamerin genes following topical application of the JH analog to virgin queens, nurses, and foragers. The four genes were down-regulated in *S*. *invicta* nurses after application of the JH analog. However, only *hexamerin 2* and *arylphorin subunit beta* were down-regulated in foragers, while *hexamerin 1* and *arylphorin subunit alpha-like* were down-regulated in virgin queens following the topical application of the JH analog. Therefore, it is possible that JH regulates hexamerins differentially in the reproductive caste than in worker caste. The reduced expression of all hexamerins in foragers compared to nurses, and the reduction of hexamerin expression following the topical application of the JH analog to nurses are consistent with the existence of a low JH titer in nurses and a high titer in foragers as shown in other ant species.

**Fig 6:**
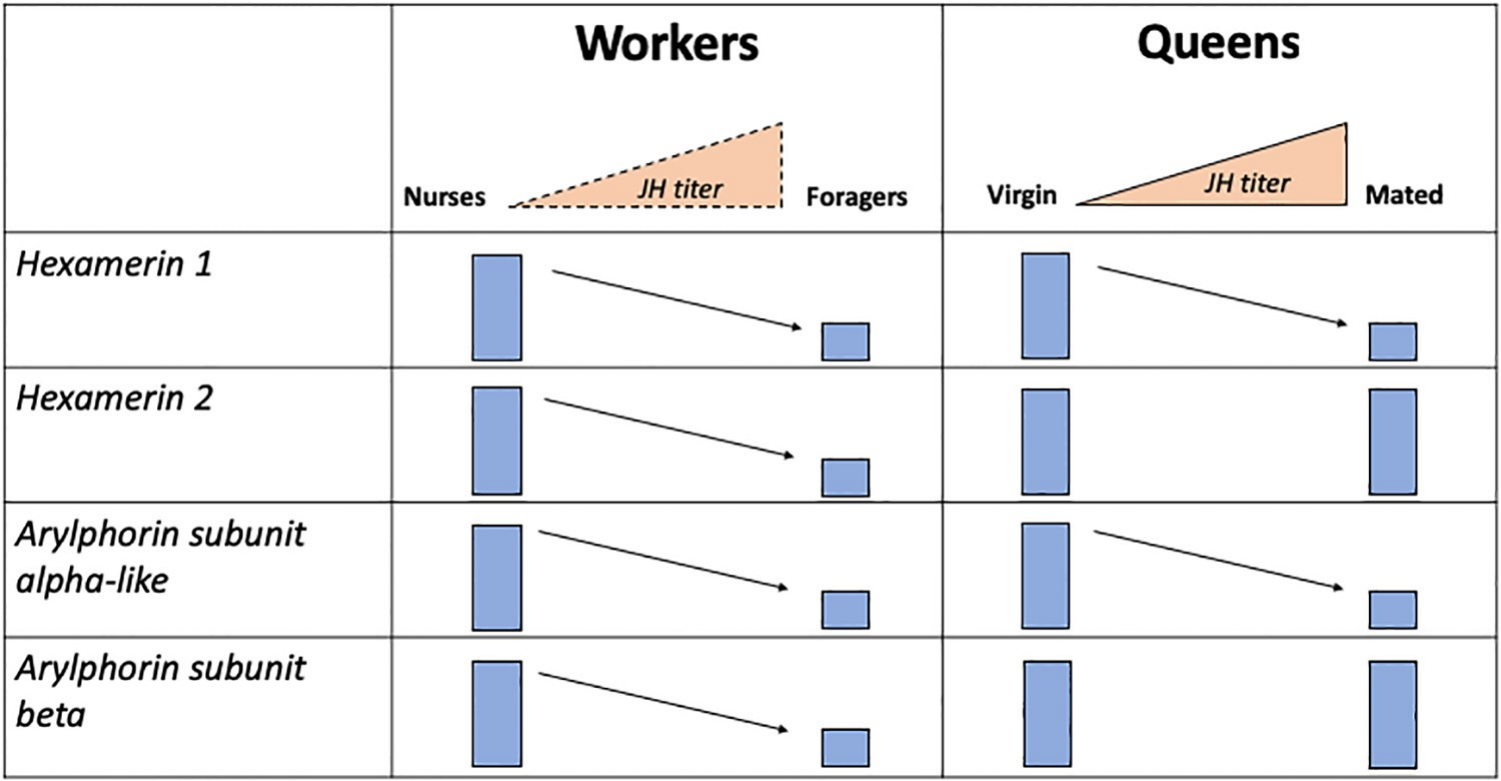
Proposed two modules regulating hexamerin expression in *S*. *invicta*: queens and workers. In workers, hexamerin expression is regulated by JH. The expression of the four hexamerins decreases as workers transition from nursing to foraging tasks, which supports the existence of changes in JH as the workers age (represented in dashed). In queens, only *hexamerin 1* and *arylphorin subunit alpha-like* are regulated by JH. Both genes are down-regulated in mated queens compared to virgin queens.

Based on the results presented in this study, we propose the existence of different regulatory modules controlling the expression of hexamerins in *S*. *invicta* in a caste-specific manner (Fig 6). In workers, JH regulates the expression of the four hexamerin genes: the four genes were down-regulated in nurses following the JH analog application and in foragers *hexamerin 2* and *arylphorin subunit beta* were also down-regulated by JH. It is possible that RT-qPCR could not detect a reduction in the expression of *hexamerin 1* and of *arylphorin subunit alpha-like* following the application of the JH analog in foragers due to low expression of these two genes in these workers. Importantly, the fact that application of the JH analog further reduced the expression of *hexamerin 2* and *arylphorin subunit beta* in medium size foragers may reflect the molecular mechanism for behavioral plasticity in medium size worker foragers; that is, increase their foraging task in response to higher levels of JH, or perhaps these ants have the ability to revert to in-nest tasks in special circumstances when JH levels may decrease.

Regulation of hexamerins by JH is different in queens. In this caste only *hexamerin 1* and *arylphorin subunit alpha-like* appear to be regulated by JH. This conclusion is based on the down-regulation of these genes but not of *hexamerin 2* and *arylphorin subunit beta* in virgin queens following the JH analog application (Fig 5). The expression analyses performed using mated and virgin queens support this finding (Fig 2). Alate virgin queens are repressed by the queen primer pheromone, and as a result they have low levels of JH, which prevent their ovaries from developing [59]. When virgin queens are released from the influence of the queen primer pheromone, their JH titers increase and as a consequence they shed their wings and their ovaries develop. The same results of wing shedding and ovary development are obtained by topical application of JH or its analogs [41,59,60,61,62]. The changes in *hexamerin 1* and *arylphorin subunit alpha-like* gene expression in alate virgin queens could be linked to the mobilization of reserves for ovary development. Additionally, these changes could also be linked to the potential involvement of hexamerins in signaling nutritional status; the down-regulation of these hexamerins could signal the end of the period in which virgin queens actively accumulate reserves.

Some hexamerins bind JH [31,63], thus it is hypothesized that these proteins might bind JH in social insects to modulate JH titers [29]. Among the *S*. *invicta* hexamerins, *arylphorin subunit alpha-like* contains a region that is the most similar to the sequence of the locust hexamerin, which if deleted, eliminates the JH binding activity [41,63]. Therefore, if the arylphorin subunit alpha-like protein has a similar expression pattern as the transcript, this protein could be involved in modulating JH titers in different *S*. *invicta* castes: in queens, it might be involved in maintaining a low JH titer in virgins to prevent precocious reproductive development [64]; while in workers, it might be involved in maintaining a low JH titer in nurses preventing transition to foraging tasks. Indeed, JH titer is low in the *A*. *mellifera* nurse and it increases as the insect ages [18,20,23,24]. While JH titers in *S*. *invicta* workers have not been measured, the results presented here support a similar role of JH in *S*. *invicta* age polyethism such as that found in other social insect species: all hexamerins were expressed at lower levels in foragers compared to nurses (Fig 2). Further, because all hexamerins were down-regulated following the application of a JH analog to nurses (Fig 4), our results also support the existence of changes in JH titers as the *S*. *invicta* worker ages.

One pitfall of the present study is that the ants used were of unknown age. The insect age could be a factor affecting the level of hexamerin expression in the alate virgin queens and the workers. The existence of age polyethism in *S*. *invicta* workers was established by Mirenda and Vinson [65] who determined that young workers stay in the nest where they perform nest-related tasks; then,they take more peripheral locations as they age, and they become forages as they near their death. While we can speculate that in the present study the foragers were older than the nurses, future studies should address the effect of age in the expression of these genes.

In conclusion, our study found a caste- and task-biased expression of hexamerins in *S*. *invicta*. Further, the regulation of the four genes by the JH analog S-hydroprene was also established, however this regulation was caste-specific for two of these genes.. While expression of these genes in *A*. *mellifera* workers might be associated with their potential for reproduction, *S*. *invicta* workers cannot reproduce. Thus, the expression of hexamerins might indicate the potential role of these genes in other aspects of *S*. *invicta* biology. In queens, *hexamerin 1* and *arylphorin subunit alpha-like* may be associated with signaling nutritional status, dominance status and/or reproductive activity. It is tempting to speculate that the lack or low level of regulation of *hexamerin 2* and *arylphorin subunit beta* in queens in response to the JH analog (Fig 5) could be pre-determined during the caste differentiation of reproductives by a mechanism involving DNA changes (methylation/demethylation), for example. Alternatively, in queens, other signaling factors have higher hierarchy to indicate the nutritional status as JH is a gonadotropin. However, it is also possible that the observed lack of regulation results from the analysis of RNA from whole body samples, thus tissue-specific analysis are needed to verify this result. In workers, hexamerin expression might be associated with nutritional signaling, task-allocation and/or age polyethism. Indeed, hexamerins in the worker caste may reflect the nutritional status of workers because foragers have the lowest levels of expression, reflecting the starved phenotype [66], and nurses, which are preferentially fed and have a higher nutritional status, have higher relative levels of all hexamerins (Fig 2). In addition, this is demonstrated in that the level of hexamerin expression in nurses can be downregulated by application of a JH analog, which would mimic the transition to foraging, again, reflecting a starved phenotype. The medium foragers, however, with lower endogenous levels of hexamerins, do not respond equally to the JH analog perhaps because the expression of two hexamerins has already decreased to a minimum level, and only *hexamerin 2* and *arylphorin subunit beta* respond to the treatment. Co-option of hexamerin genes may have given *S*. *invicta* colony the ability to respond to cues involved in social organization and queen dominance, and the expression of these storage proteins in adults may be based on colony demands. The gene expression analyses presented here illustrates the potential for hexamerins to play important roles in fire ant social physiology, warranting protein level analysis and reverse genetic manipulation to further elucidate the evolutionarily coopted roles of these genes/proteins in relation to the reproductive ground plan hypothesis.

## Supporting information

S1 Fig Constructed cages for treatment groups of queens: (A) Materials used for the cage are a 15 mL tube, fine netting cut into a square, parafilm to hold down the netting. (B) Constructed treatment cage. (C) After treatment alate queens were put into tubes and lid fastened. (D) Cages were put back into the colony. (E) 100% of alate queens from the S-hydroprene treatment dealated.
(TIFF)

S2 Fig Alignment of the four *S*. *invicta* proteins using MAFT. () indicates non conservative mutations, (*) indicates conserved regions, (:) indicates conservative replacement mutations and (.) indicates semi conservative mutations.
(DOCX)

S1 Table Primers used for qRT-PCR analysis. (DOCX)
