# Supplementary figures and images for: Caste-based differential transcriptional expression of hexamerins in response to a juvenile hormone analog in the red imported fire ant (Solenopsis invicta)
Source: PLoS One. 2019 May 20;14(5):e0216800. doi: 10.1371/journal.pone.0216800 (PMC6527210; doi:10.1371/journal.pone.0216800)

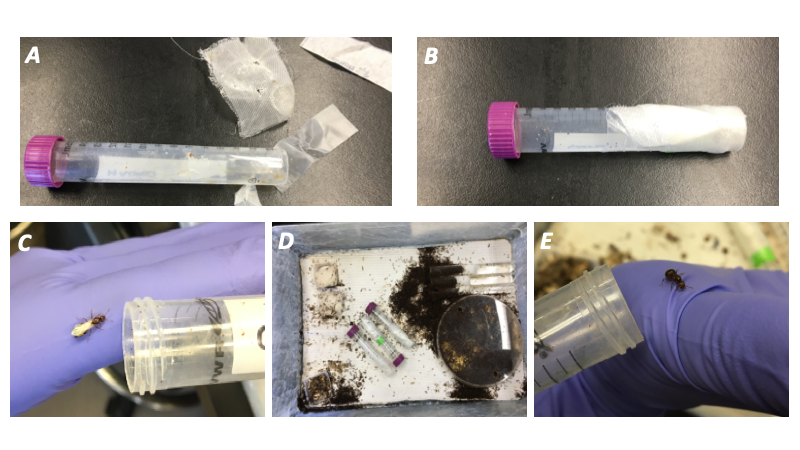

Supplement: S1 Fig — Constructed cages for treatment groups of queens: (A) Materials used for the cage are a 15 mL tube, fine netting cut into a square, parafilm to hold down the netting. (B) Constructed treatment cage. (C) After treatment alate queens were put into tubes and lid fastened. (D) Cages were put back into the colony. (E) 100% of alate queens from the S-hydroprene treatment dealated. (TIFF) [file pone.0216800.s001.tiff]

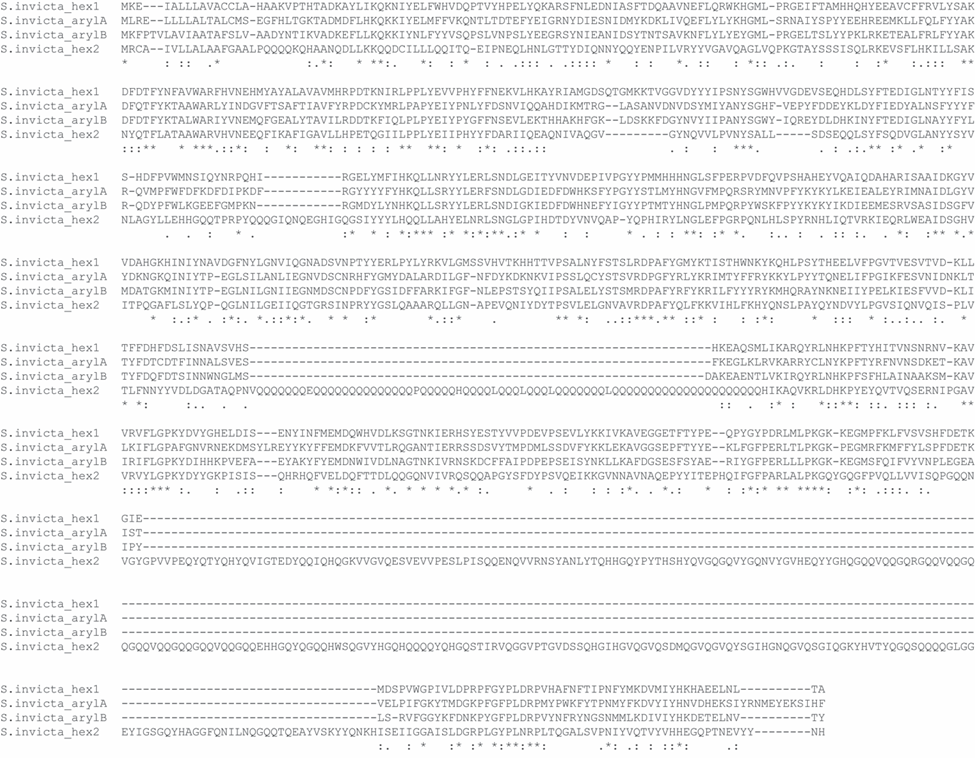

Supplement: S2 Fig — () indicates non conservative mutations, (*) indicates conserved regions, (:) indicates conservative replacement mutations and (.) indicates semi conservative mutations. (DOCX) [file pone.0216800.s002.docx]
