# Supplementary material for: Caste-based differential transcriptional expression of hexamerins in response to a juvenile hormone analog in the red imported fire ant (Solenopsis invicta)
Source: PLoS One. 2019 May 20;14(5):e0216800. doi: 10.1371/journal.pone.0216800 (PMC6527210; doi:10.1371/journal.pone.0216800)
Supplement: S1 Table — (DOCX) [file pone.0216800.s003.DOCX]

Supplementary

S1. Primers used for qRT-PCR analysis

| Gene | Forward primer 5’-3’ | Reverse primer 5’-3’ | Product (bp) |
| --- | --- | --- | --- |
| Hexamerin 1 | GGGTGTTACGGTCGAATCTG | TGAGCCTCCTTGTGACTGTG | 108 |
| Hexamerin 2 | CGTAGGATATGGACCGGTTG | GCCTTGATGTTGGATTTGCT | 97 |
| Arylphorin-Alpha | GCCCTTTCCGTAGAGAGCTT | TGTTGACGTTGAAGCGGTAG | 103 |
| Arylphorin-Beta | GCCGACTACAACACCATCAA | CGAAGGATTCACCAGAGAGG | 108 |
| RPL18 | TACACCGACCACCGATTTCA | GATCACGGCGACGCAATT | 80 |
